# Characterization of partially ordered states in the intrinsically disordered N-terminal domain of p53 using millisecond molecular dynamics simulations

**DOI:** 10.1038/s41598-020-69322-2

**Published:** 2020-07-24

**Authors:** Pablo Herrera-Nieto, Adrià Pérez, Gianni De Fabritiis

**Affiliations:** 1 Computational Science Laboratory, Barcelona Biomedical Research Park (PRBB), Universitat Pompeu Fabra, C Dr Aiguader 88, 08003 Barcelona, Spain; 2 Institució Catalana de Recerca i Estudis Avançats (ICREA), Passeig Lluis Companys 23, 08010 Barcelona, Spain

**Keywords:** Peptides, Proteins, Intrinsically disordered proteins

## Abstract

The exploration of intrinsically disordered proteins in isolation is a crucial step to understand their complex dynamical behavior. In particular, the emergence of partially ordered states has not been explored in depth. The experimental characterization of such partially ordered states remains elusive due to their transient nature. Molecular dynamics mitigates this limitation thanks to its capability to explore biologically relevant timescales while retaining atomistic resolution. Here, millisecond unbiased molecular dynamics simulations were performed in the exemplar N-terminal region of p53. In combination with state-of-the-art Markov state models, simulations revealed the existence of several partially ordered states accounting for $\sim$ 40% of the equilibrium population. Some of the most relevant states feature helical conformations similar to the bound structure of p53 to Mdm2, as well as novel $\beta$-sheet elements. This highlights the potential complexity underlying the energy surface of intrinsically disordered proteins.

## Introduction

Over the last decades the understanding of protein function was summarized by the *sequence-structure-function* triumvirate: protein sequences encode folds able to perform specific tasks. Intrinsically disordered proteins (IDPs) defy this principle by mediating their biological functions despite lacking a stable three-dimensional structure^1–3^. Such behavior configures a relatively flat energy surface where many isoenergetic conformations coexist^4^. This surface can be modified to a certain extent, as revealed by the shift towards certain subpopulations observed in the formation of protein-IDP^5–8^ or molecule-IDP complexes^9,10^. Similarly, kinetic parameters governing the conversions amongst subpopulations can also be modified by post-translational modifications^11,12^. Thus, the energy surface of IDPs if far from being constituted exclusively by random coiled conformations, and pieces of evidence support the existence of partially ordered states^11^. The characterization of such partially ordered states is crucial to understand IDPs’ function, their mechanisms of action, and their potential modulation.

The structural heterogeneity of IDPs is summarized as a collection or ensemble of conformations. They can be resolved experimentally by using nuclear magnetic resonance (NMR) or small-angle X-ray scattering data. The main limitation of IDP ensembles resolved in that way is that they focus on global averages rather than diving in particular atomic coordinates^13^. There are also many computational approximations to address this task^14^. They generally involve an initial step of conformer generation followed by a refinement step that minimizes differences between the generated library and experimental data. However, many computationally resolved ensembles can match the same experimental observations.

Molecular dynamics simulations (MD) have been extensively used over the years to navigate complex energy surfaces in other biological problems, i.e. in folding^15^, protein–protein binding^16,17^ and, modulation of IDPs by post-translational modifications^11^ or by interacting with their folded partners^8^. In the context of IDP ensembles, MD simulations have been primarily applied as a tool for conformational generation. Nevertheless, the main goal of MD in this area would be to define reliable ensembles without the need for biasing or reweighting procedures. In this line, recent studies have employed enhanced sampling methods such as Hamiltonian replica exchange MD to define IDP ensembles matching the available experimental information^18^. In terms of aggregated time, the study run for $\sim$ 10 $\mu s$, while others have performed more extensive simulations, $\sim$ 200 ${\upmu }{\text{s}}$ but in a single trajectory^19^.

Current technologies allow MD simulations to reach aggregated times in the order of milliseconds^20^, thus making this tool a valuable one for the exploration of biological systems at increasingly longer time scales^16^. The potential offered by high throughput MD simulations coupled with Markov State Models (MSMs)^21^ analysis for the exploration of conformational landscapes of IDP has been tested in some aggregation-prone peptides^22,23^. The main advantage offered by this tandem is the possibility to address subpopulations within ensembles and to study the kinetics controlling them, rather than working with population averages. By focusing on the most relevant subpopulations and their kinetic properties, it is possible to gain insight into the emergence of partially ordered states in atomistic detail.

Here we make use of extensive, unbiased full-atom MD simulations and state-of-the-art MSMs to explore the structural variability of the N-terminal region of p53 in isolation. p53 is a widely studied protein, given its relation to oncogenic processes. It includes disordered sections at both N and C terminals, which interacts with various partners^24^. Of special importance is the short region comprising residues 10 to 40. Its forms a stable $\alpha$-helix upon interaction with the Mdm2 protein^5^ and has been the primary subject for many computational and experimental studies. The p53–Mdm2 complex has served as a template for the development of peptidomimetics drugs^25^ and as the preferred benchmark for several MD studies aiming to reconstruct the binding process and the associated kinetics^16,26–29^. NMR and SAXS studies of the N-terminal region in isolation revealed a helicity profile similar to the one observed in the p53-Mdm2 complex, implying that bound conformations might also be sampled prior to binding^30^.

The main results show the existence of many kinetically relevant states, accounting for $\sim$ 40% of the equilibrium population, including high levels of secondary structural elements. In particular, simulations show the presence of an $\alpha$-helix enriched states similar to the folded pose found in complex with Mdm2, as well as, a tangled interplay between $\beta$-strands formation leading to novel $\beta$-sheet enriched structures. Altogether, this illustrates the complexity of partially ordered states within the conformational space of an exemplar IDP, such as the N-terminal region of p53.

## Results and discussion

### Identification of secondary structure enriched states

**Figure 1:**
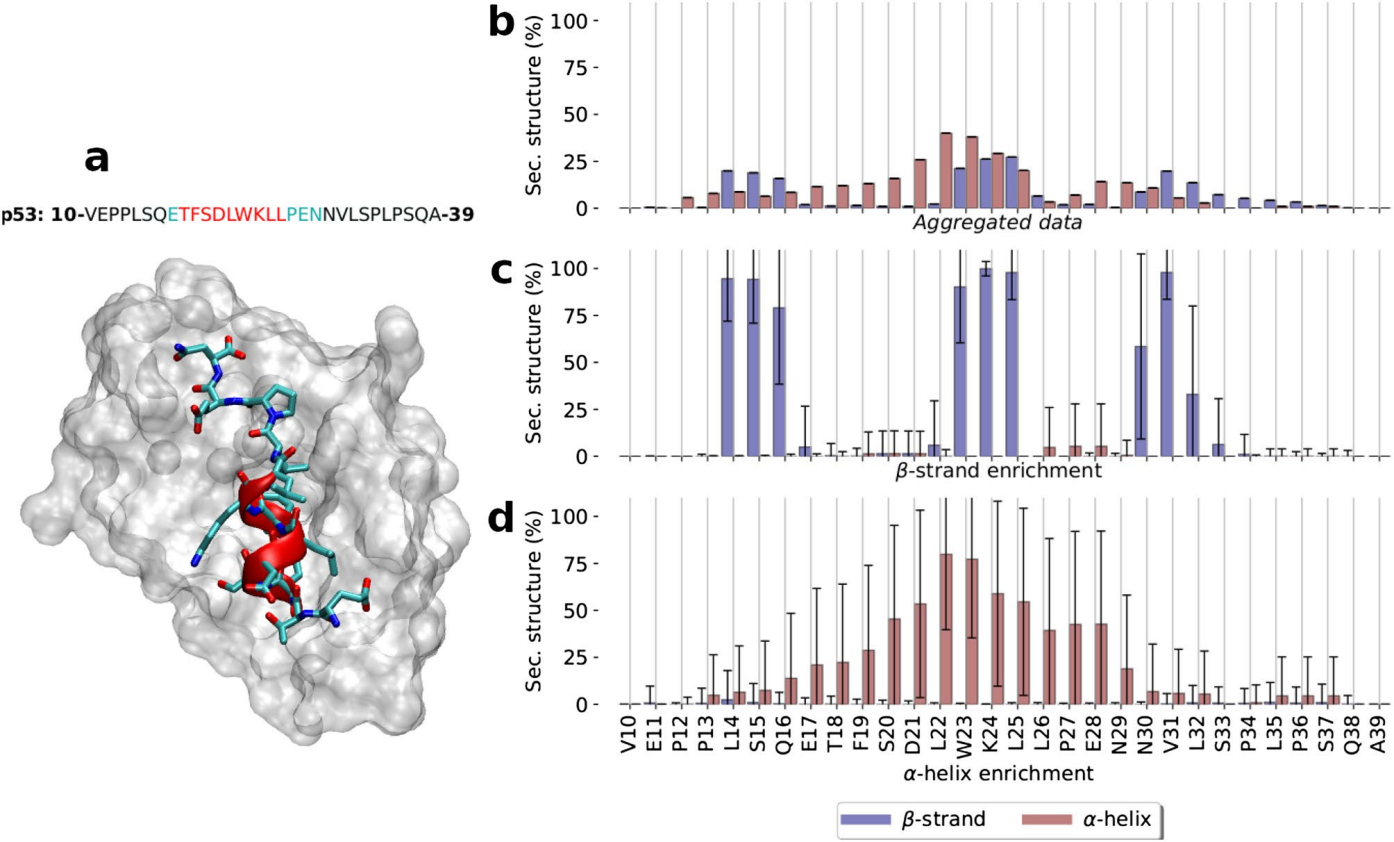
p53 secondary structure propensities (**a**) p53–MDM2 complex: MDM2 protein is shown as a white surface. p53 is depicted as cyan sticks and the helical region between residues 18 and 26 as red cartoon, (PDB code: *1YCR*). Above, the sequence of p53 used for the simulation is displayed: in red the helical section, in cyan the rest of the peptide found in the PDB structure, and in black the extended sequence. Secondary structure profiles derived from MD data: $\beta$-strand and $\alpha$-helix profiles for the (**b**) aggregated data and for those macrostates of the MSM enriched in either (**c**) $\beta$-sheet and (**d**) $\alpha$-helix.

The simulation time of the MD run totaled $\sim$ 1.4 ms. Initially, the secondary structure of the aggregated MD was analyzed. Data showed the coexistence of both $\alpha$-helix and $\beta$-strand, each one peaking at $\sim 20\ \%$ in the central region of the protein (Fig. 1b). The helicity profile follows a bell-shaped distribution, while $\beta$-strand is more sparsely scattered in three groups in the proximity of residues S15, K24, and V31.

MD data was used to create an MSM based on $backbone_{C\alpha }+sidechain_{O,N}$ self distance matrix that splits the space into 11 different sets of kinetically related conformations referred to as macrostates (labeled as *M1-11*). MSM subpopulations successfully separate metastable sets of conformations enriched in each secondary structure type (Fig. 1c,d), implying that these structural elements do appear in a concerted way, rather than being the average of residue independent structural propensities.

The helicity profile displayed by the helix-enriched state matches the bound conformation of p53 when interacting with Mdm2 (Fig. 1a). It spams from residue T18 to L26, and maximum levels of helicity being found in W23—an essential amino acid for that interaction. Similar profiles arise from NMR studies^30^. Besides this state, many others also display various degrees of helicity (Fig. S1). The tendency of IDPs to acquire secondary structure profiles resembling their folded conformation has also been observed in other IDPs, and it has also been related to the binding mechanism to their partner^31^ and their signaling properties^32^.

For $\beta$-strand, segregation of secondary structural elements into their own states becomes especially evident in *M2*, where three $\beta$-strands—namely $\beta$1, $\beta$2, and $\beta$3 from N to C terminal—are organized in an anti-parallel double-sheet (Fig. 1c), defining the partially ordered state with the most significant level of structure. Besides the secondary structure enriched macrostates aforementioned, many other states also exhibit different profiles of $\beta$-strand and $\alpha$-helix (Figs. S1 and S2). This includes a number of states displaying different $\beta$-sheet arrangements, featuring only one strand, either $\beta$1-$\beta$2 or $\beta$2-$\beta$3. Altogether, this highlights the variety of possible configurations found across the conformational landscape of p53.

### Kinetic characterization of the conformational landscape of p53

**Figure 2:**
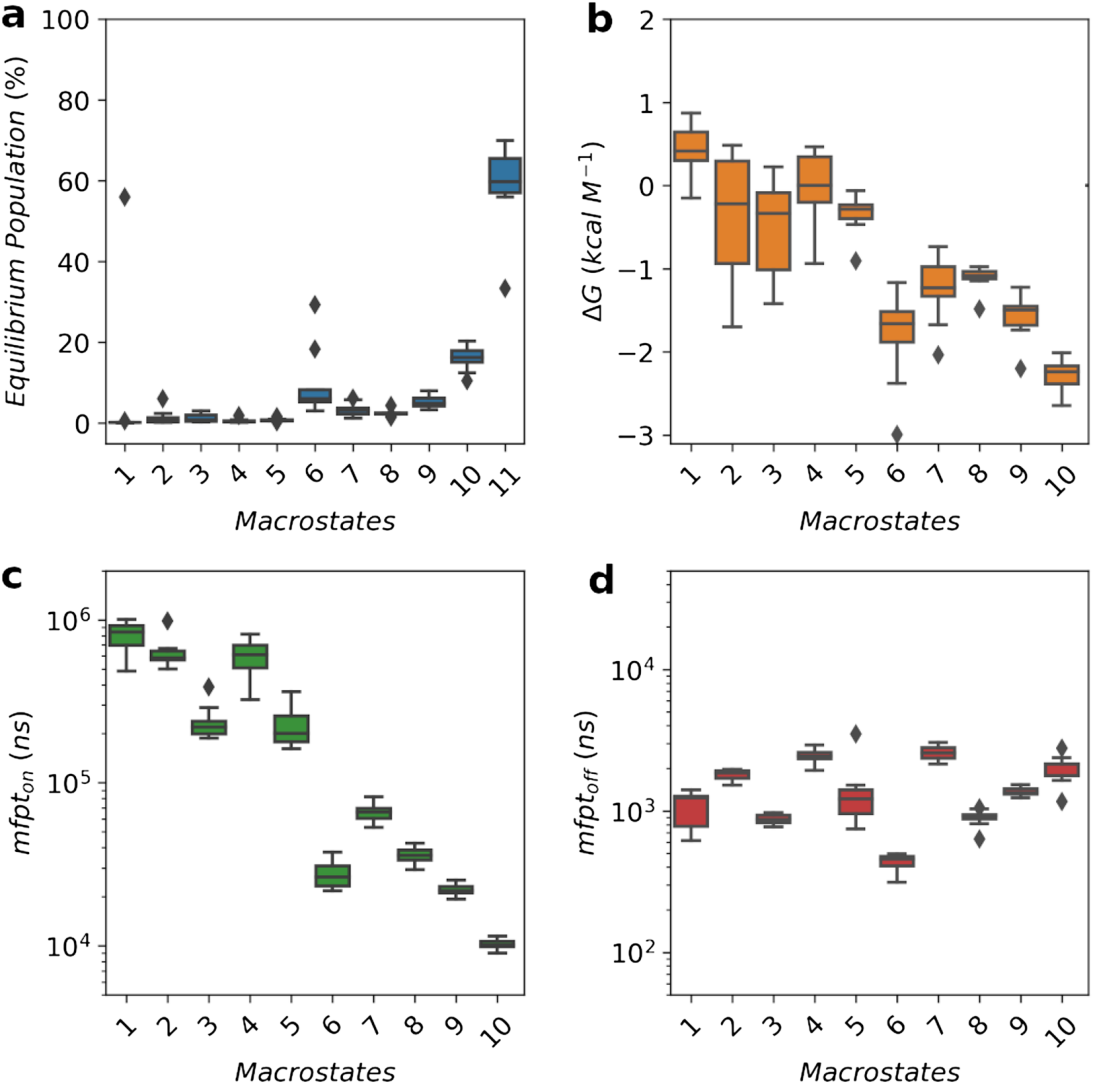
Estimation of (**a**) $equilibrium\ population$, (**b**) $\Delta G$, (**c**) $mfpt_{on}$, (**d**) $mfpt_{off}$, for each macrostate after 10 rounds of bootstrap. Kinetics parameters for *M11* (structurally heterogeneous) are not shown as it was used as the source state for calculations.

Population wise, partially ordered states account for a significant proportion of the equilibrium population ($\sim$ 40%, Fig. 2a). The triple-stranded macrostates, the most folded ones, have low populations (< 1%), in contrast to double-stranded states like *M10*, which reach $\sim$ 20% at equilibrium. However, the most populated state—*M11*; with $\sim$ 60% of the population—is structurally heterogeneous, and lacks any secondary structural element or long-range contacts. Hence, partially ordered states are not energetically favored compared to the most extended configurations, and their free energies range from 0.5 to − 2.5 k cal $M^{-1}$ (Fig. 2b). This can be visualized in more detail in the energy surface of p53 (Fig. S3). There are two well-defined minima separated by a small energy barrier. One of them is covered with the extended and the helical states (*M6,9,11*), and the other by $\beta$2–$\beta$3 conformations (*M10*). High energetic areas are occupied by the most structured states, like *M2*. Such profile, with many energetically similar states, fits the description of IDPs in isolation. The level of compaction of p53 follows a similar trend (Fig. S4). The most collapsed states have a radius of gyration close to the expected for a folded protein. More flexible ones combine rigid and non-rigid sections and sample extended conformations. The lack of over-compaction of the ensemble is in line with the capabilities of state-of-the-art force fields^33^.

The distinctive population profiles previously observed have an impact on the kinetic behavior of the macrostates. There is an approximately two orders of magnitude difference between maximum and minimum $mfpt_{on}$ estimations, which separate *faster/more populated (M6-10)* macrostates from *slower/less populated (M1-5)* macrostates (Fig. 2c). *Faster* macrostates comprise the helical conformation and several double-stranded states. *Slower* states, on the other hand, include triple stranded states and other low populated states. Off rates remain similar in all macrostates (Fig. 2d).

**Figure 3:**
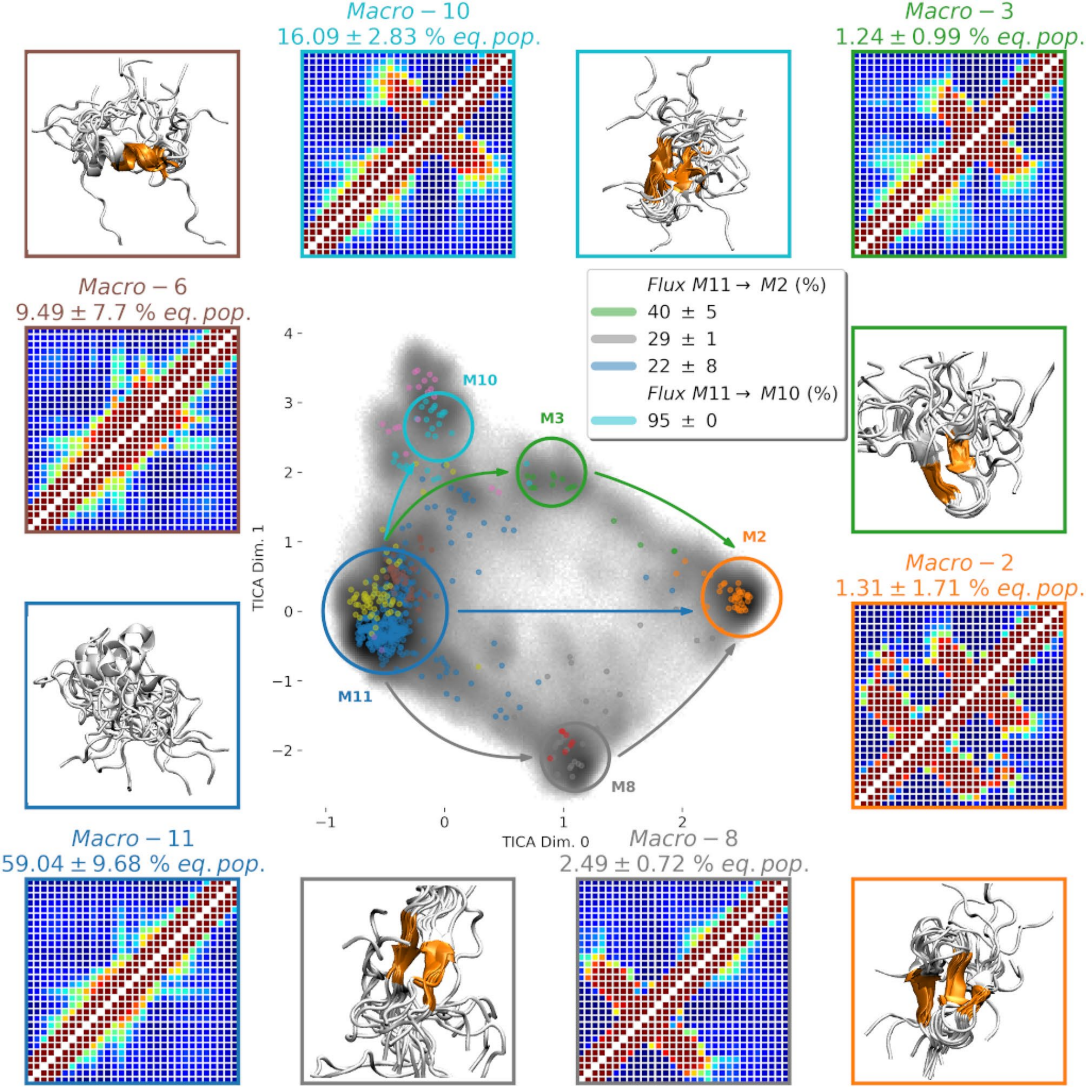
p53 conformational landscape. Central panel illustrates the first two dimensions of the TICA space. In grey, a 2D histogram $200 \times 200$ bins represents the frame count of the aggregated MD data. MSM microstates are distributed accordingly to their centers and colored with respect to their corresponding macrostate. Arrows represent the main pathways leading from the most extended macrostate (*Macro-11*) to the most $\beta$-sheet enriched ones (*Macro-2* and *Macro-10*). Correspondence between macrostate location in the central panel and side panels is color mapped. Side panels describe macrostates in terms of residue-residue contacts maps. Protein visualization is performed by superimposing 20 structures using residues highlighted in orange for structural alignment.

We employed transition path theory^34,35^ to study the most relevant pathways and fluxes for macrostate interconversion. In particular, the focus was to elucidate the folding process leading from the less structured state to the triple-stranded conformation. There are three main paths (Fig. 3—central panel) involved in this process. The least transited one accounts for $\sim$ 15% of the total flow, and directly reaches the folded conformation from the extended one. On the other hand, the most transited paths involve the participation of double-stranded intermediates, with the $\beta$1–$\beta$2 structure taking $\sim$ 40% and the $\beta$2–$\beta$3 conformation being responsible for the remaining $\sim$ 30% of the flux. Additionally, other $\beta$-enriched states, such as the extended $\beta$2–$\beta$3 sheet found in *M10*, are disconnected from this network and can be directly reached from *M11* without the need of intermediates. It is interesting to point out that some conformations (such as *M3,10*), despite their structural similarity, show a $\sim$ 30 slowdown that explains the differences in stability aforementioned.

In summary, partially ordered states populating the conformational landscape are structural and kinetically diverse. States coexist at different timescales, even if they are structurally similar, such as the case of *M6,11*. These two states feature a short and an extended $\beta$2-$\beta$3 sheet but have $k_{on}$ values of $5 \cdot 10^{5} \;{\text{M}}^{{ - 1}} \;{\text{s}}^{{ - 1}}$ and $1 \cdot 10^{7} \;{\text{M}}^{{ - 1}} \;{\text{s}}^{{ - 1}}$ respectively.

### Comparison with NMR data

In order to ensure and validate MD observations, simulation data were compared against experimentally determined backbone chemical shifts (CS) for the N-terminal region of p53^36^. Experimental CS were resolved for the full-length N-terminal (residues 1–93), but only CS for residues 10 to 39 were used (to match the simulated sequence). CS allows inferring by-residue secondary structure tendencies on folded and disordered proteins. Calculations were performed using two softwares, SPARTA+^37^ and SHIFTX2^38^, on a set of 2 000 structures selected at random accordingly to the macrostate equilibrium probabilities. Calculated CS with both programs yielded similar results. Overall, there is a high correlation between experimental and MD calculated CS values for C$\alpha$, C$\beta$, and N with $R^2$ values of 0.98, 0.99, and 0.88 (Fig. S5). Differences between experimental and calculated CS remains within the intrinsic estimations error of each software ($\sim 1\ \text{p.p.m}$ for SPARTA+ and 0.4, 0.5 and 1.1 for C$\alpha$, C$\beta$ and NH, respectively, for SHIFTX2) thus, indicating that structural rearrangements observed in MD data are in line with those determined by NMR experiments.

## Conclusion

The characterization of the p53 conformational landscape using unbiased MD simulations revealed a high number of transient partially ordered states accounting for $\sim$ 40% of the equilibrium populations. Partial order arises from the formation of both $\alpha$-helix and $\beta$-strand structural elements. The helical state resembles the structure acquired by p53 upon interaction with Mdm2. The MSM also showed the presence of several $\beta$-enriched states, not described before, that established long-range contacts through the arrangement of either one or two $\beta$-sheets. These processes are kinetically different, and some of the faster states are quickly accessible from the random-coiled macrostate and highly populated at equilibrium. Thus, it would be possible for some of them to play biologically relevant roles and could even provide novel strategies for the modulation of IDPs. Other computational studies with p53^39^ also hinted the presence of collapsed conformations in this region. The exploration of aggregation-prone IDPs, such as the amyloid beta^22^ and hIAPP^23^, showed the spontaneous formation of $\beta$-hairpin metastable states, but with very small populations.

The current study provides a structural and kinetically detailed description of the conformational landscape of an IDP using MD simulations in combination with MSMs. Given the high number of short linear motifs within the human proteome, a similar pipeline could, in principle, be more extensively applied in order to investigate whether other IDPs may also share such complex behaviors. However, reaching millisecond simulation time may not prove scalable in more extensive studies with multiple targets. Novel adaptive sampling techniques^40,41^, which perform a more intelligent exploration of surfaces, might mitigate this problem by reducing the computational time needed to achieve similar results. Some final considerations about the task is the computational effort to simulate longer peptides as well as the need to compare with experimental data to validate partially folded states and their relaxation times. Here we use a relatively short 30 amino acids section of p53, while completely disordered domains may spam hundreds of residues.

## Methods

### Molecular dynamics simulation set up

In order to perform the exploration of the conformational space of p53, extensive parallel simulations were run. The selected region of p53 spammed from residue 10 to 39.

A set of 110 structures was used as initial conformations for the MD run (Fig. S8c). All systems were built with VMD^42^ (version 1.9.2, http://www.ks.uiuc.edu/Research/vmd/), solvated with TIP3P^43^ (each system included $\sim$ 8,200 water molecules, resulting in a final protein concentration of $\sim$ 6.8 mM), with a final NaCl concentration of $0.05\ \text{M}$. A Langevin integrator with a damping constant of 0.1 $\text{ps}^{-1}$ was used. The integration step was set to 4 fs, with heavy hydrogen atoms scaled up to four times their natural mass. Electrostatic were computed using PME with a cutoff distance of 9 Å and grid spacing of 1 Å. Equilibration was performed at 300 K, firstly undergoing 250 steps of energy minimization followed by 0.1 ns simulations in an NVE ensemble (pressure was kept at 1 atm by using the berendesen barostat) and 2 ns in an NPT ensemble. We employed the CHARMM22* forcefield^44^, a modification of the original CHARMM22 with adjusted backbone torsion potentials to produce more extended conformations. After equilibration, no proline *cis* isomers were detected.

Production runs of 1 $\upmu s$ were performed at 310 K using the distributed computing project GPUGrid^45^ using the ACEMD engine^46^ (included in HTMD).

### Markov state model analysis

Production runs generated a total of 1.337 trajectories (each equilibrated system was used at least 10 times) of $1\, {\upmu }{\text{s}}$ each, similarly to^16^. Thus, the production runs accounted for an aggregated simulation time of $\sim$ 1.4 ms in order to maximize the exploration of the conformational space. All MD data analyses were performed using HTMD^47^ (version 1.22.0 https://github.com/Acellera/htmd).

MD data was used to build a MSM. The analysis was performed by featurizing atomic coordinates as the self-distance matrix between $C_{\alpha }$ and side chains nitrogen and oxygen atoms. Next, time independent component analysis method (TICA^48^) reduced data dimensionality at a fixed lag time of 20 frames. The parameters for the last building stages were selected based on the generalized matrix Rayleigh quotient (GMRQ) scores (Figs. S6 and S7). A final number of 9 TICA dimension and 600 clusters were selected. Data clusters were defined using the MiniBatchKMeans algorithm^49^.

Microstates were fused at a lag time of 120 ns, following the implied time scales plot (Fig. S8a) into 11 macrostates (using the PCCA+ algorithm^50^ and based on the discretization of the TICA space shown in Fig. S8e). The Chapman–Kolmogrov test (Fig. S9) confirmed that parameters yield a markovian model. Finally, transition path theory^34,35^ was used to calculate fluxes between states.

For every measure, the error was estimated by creating 10 independent bootstrap replicas of the MSM using a random set containing 80% of the trajectories.

### Chemical shift calculations

Calculations of MD derived chemical shifts were performed using 2,000 frames distributed amongst macrostates based on their equilibrium probability. The biological magnetic resonance data bank entry *17760*^36^ was used to obtain experimental chemical shift data for the N-terminal region of p53. The experiment was performed with the full-length N-terminal (residues 1 to 93), but for the comparison we used the section 10–39. Two different softwares were used: SPARTA+^37^ (version 2.90, http://spin.niddk.nih.gov/bax/software/SPARTA+/index.html) and SHIFTX2 (version 1.09, http://www.shiftx2.ca/)^38^.

## Supplementary Information

Supplementary information
