# Author Correction: Characterization of partially ordered states in the intrinsically disordered N-terminal domain of p53 using millisecond molecular dynamics simulations

**DOI:** 10.1038/s41598-020-72315-w

**Published:** 2020-09-29

**Authors:** Pablo Herrera-Nieto, Adrià Pérez, Gianni De Fabritiis

**Affiliations:** 1 Computational Science Laboratory, Barcelona Biomedical Research Park (PRBB), Universitat Pompeu Fabra, C Dr Aiguader 88, 08003 Barcelona, Spain; 2 Institució Catalana de Recerca I Estudis Avançats (ICREA), Passeig Lluis Companys 23, 08010 Barcelona, Spain

Correction to: *Scientific Reports* 10.1038/s41598-020-69322-2, published online 24 July 2020

This Article contains a repeated typographical error, where the word “span” is incorrectly written as “spam”.

As a result, in the Results and discussion section, under the subheading ‘Identification of secondary structure enriched states’,

“It spams from residue T18 to L26, and maximum levels of helicity being found in W23—an essential amino acid for that interaction. Similar profiles arise from NMR studies^30^.”

should read:

“It spans from residue T18 to L26, and maximum levels of helicity being found in W23—an essential amino acid for that interaction. Similar profiles arise from NMR studies^30^.”

In the Conclusion,

“Here we use a relatively short 30 amino acids section of p53, while completely disordered domains may spam hundreds of residues.”

should read:

“Here we use a relatively short 30 amino acids section of p53, while completely disordered domains may span hundreds of residues.”

In the Methods, under the subheading ‘Molecular dynamics simulation set up’,

“The selected region of p53 spammed from residue 10 to 39.”

should read:

“The selected region of p53 spanned from residue 10 to 39.”

