# Supplementary material for: Characterization of partially ordered states in the intrinsically disordered N-terminal domain of p53 using millisecond molecular dynamics simulations
Source: Sci Rep. 2020 Jul 24;10:12402. doi: 10.1038/s41598-020-69322-2 (PMC7382488; doi:10.1038/s41598-020-69322-2)
Supplement: Supplementary file 1 — Supplementary information [file 41598_2020_69322_MOESM1_ESM.pdf]

Supplementary Information: Characterization of partially ordered states in the p53 intrinsically disordered N-terminal domain using millisecond molecular dynamics simulations

Pablo Herrera-Nieto[a], Adrià Pérez[a], and Gianni De Fabritiis[a,b]

[a]Computational Science Laboratory,Barcelona biomedical research park (PRBB), Universitat Pompeu Fabra, C Dr Aiguader 88, Barcelona 08003, Spain
[b]Institució Catalana de Recerca i Estudis Avançats (ICREA), Passeig Lluis Companys 23, 08010 Barcelona, Spain

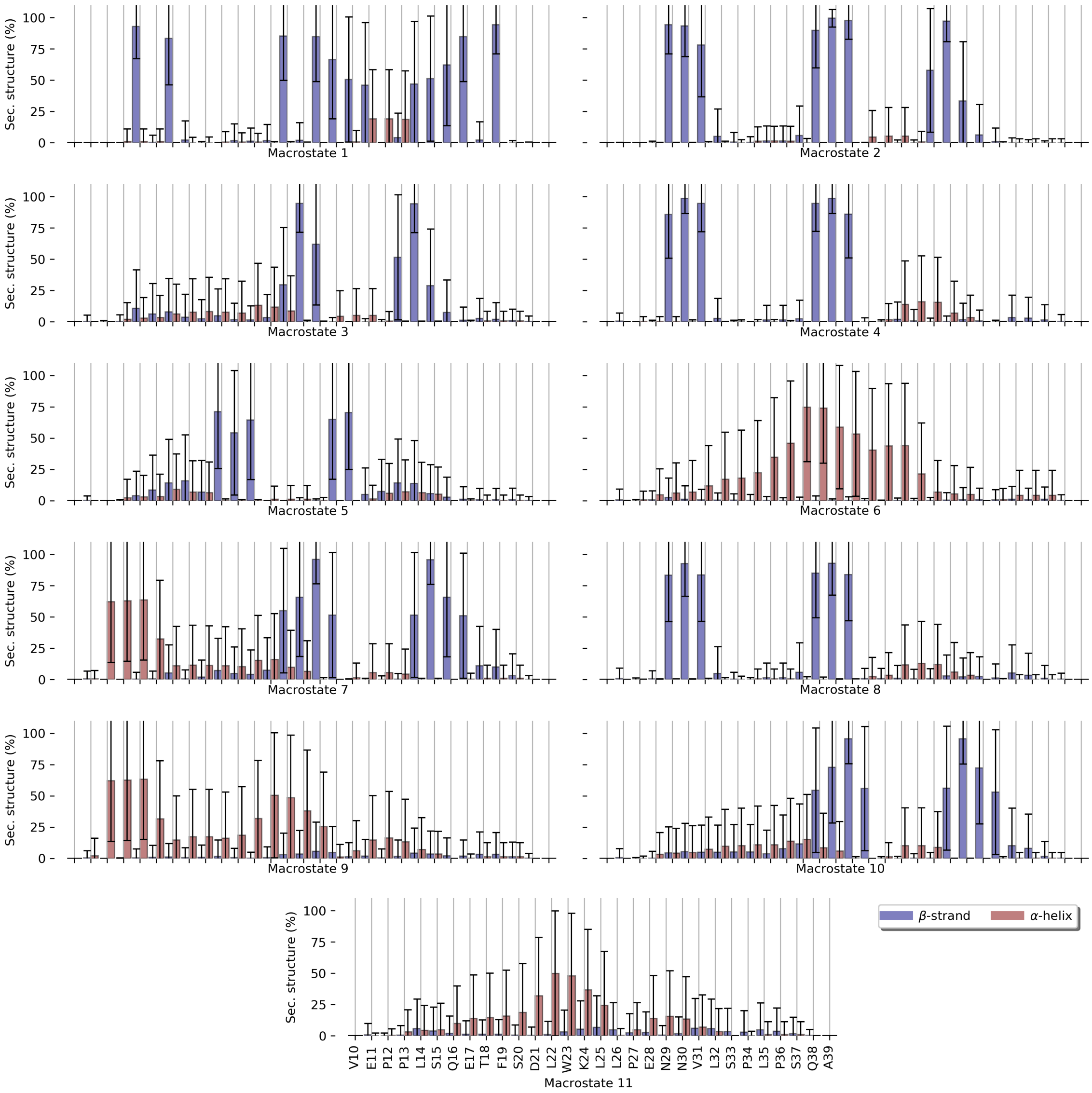

Figure 1: **Secondary structure profile** for all MSM macrostates.

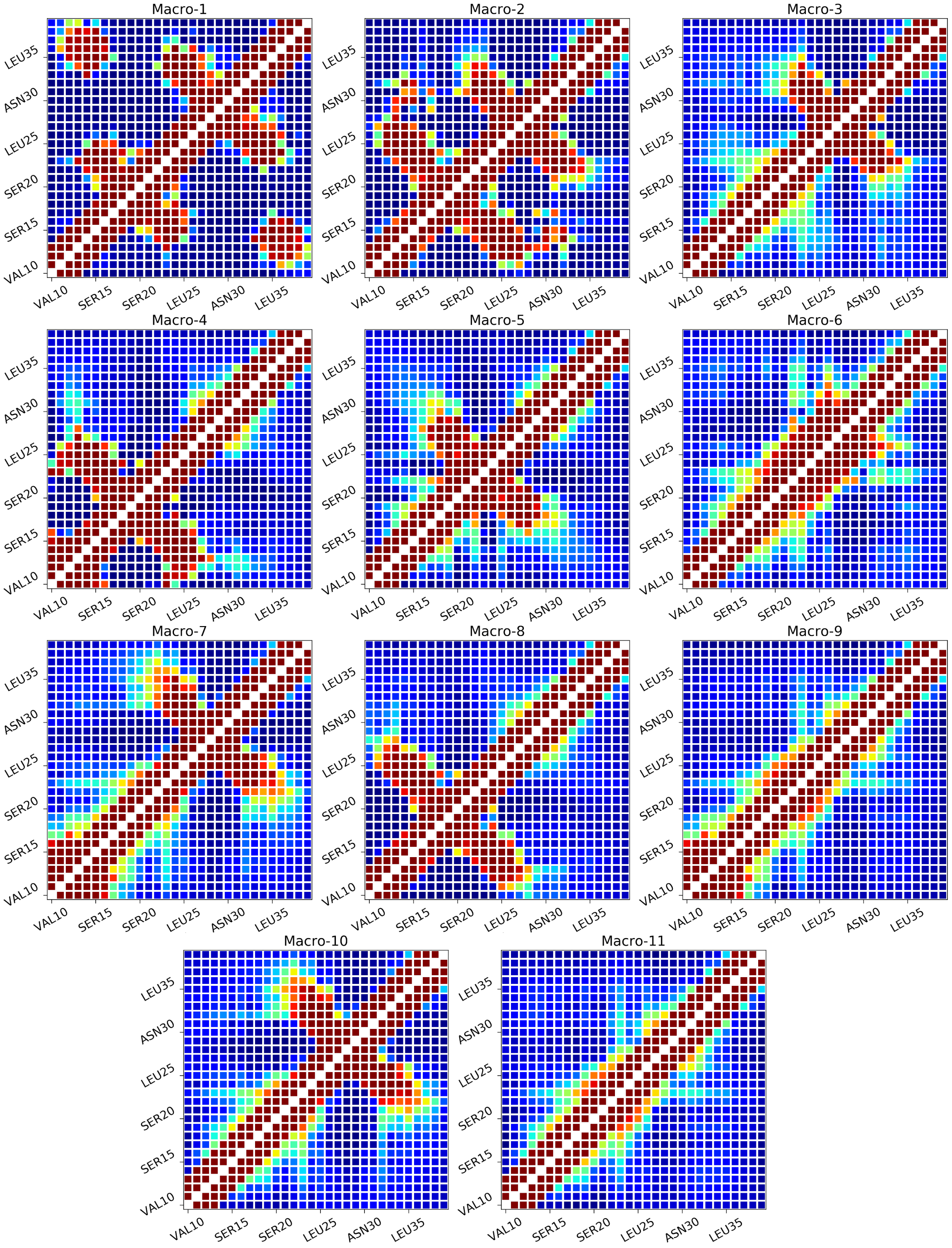

Figure 2: **Residue-residue contacts** for all MSM macrostates.

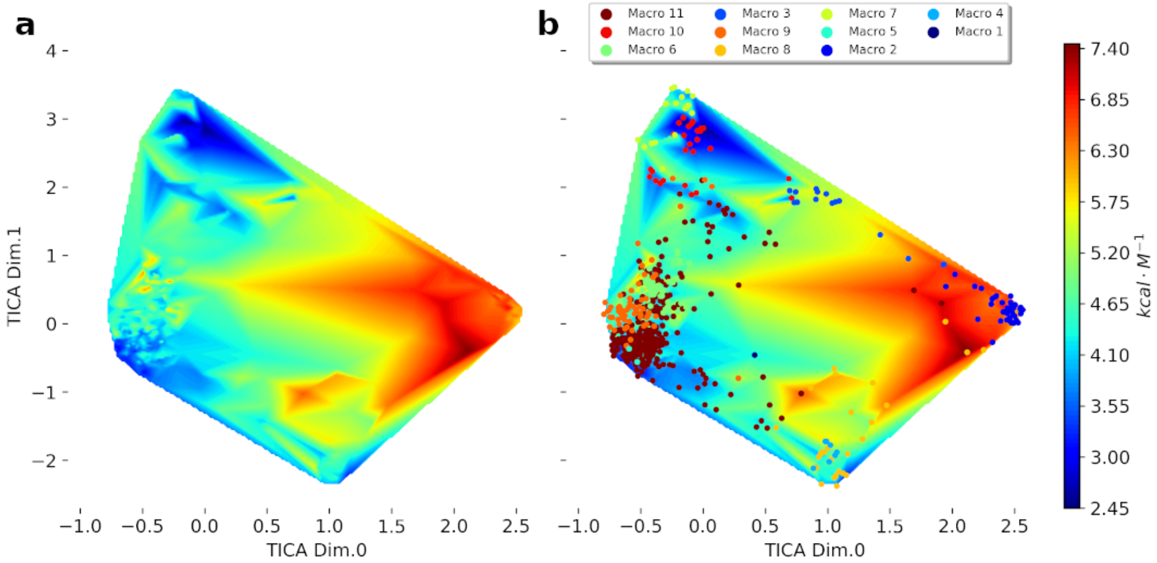

Figure 3: **Free Energy surface** of p53 in **a)** p53, and microstates centers in **b)**. Microstates are color mapped according to their macrostates.

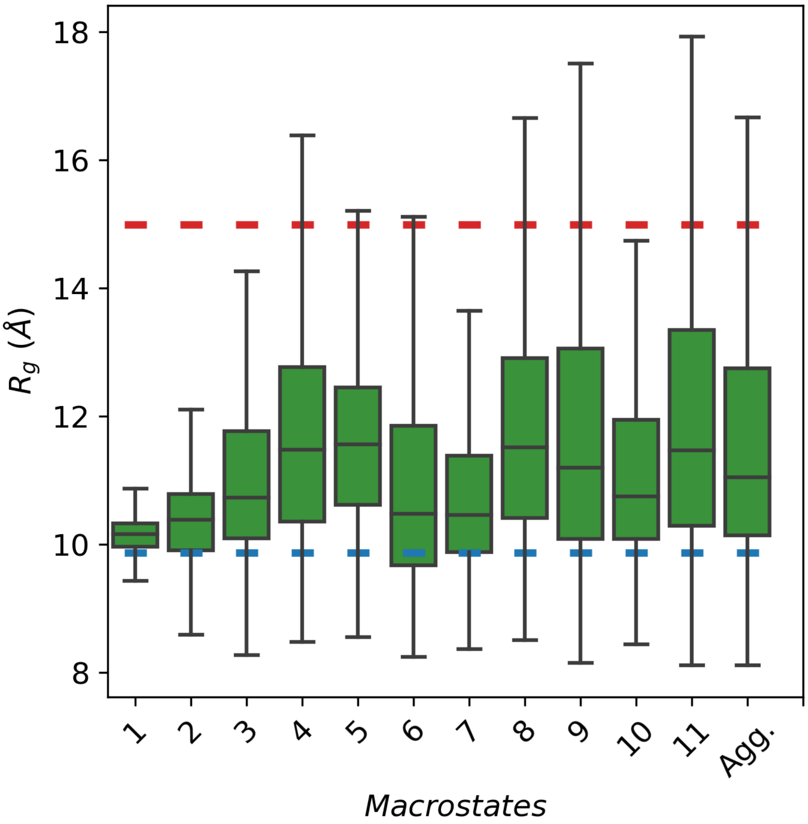

Figure 4: **Radius of gyration** (Rg) by macrostate. Rg was computed as $Rg = \sqrt{\sum_{i=1}^{n} m_i * s_i^2 / \sum_{i=1}^{n} m_i}$ for a molecule of n atoms, where s_i is the distance to the center of mass of each atom. Theoretical upper (in red) and lower (in blue) bounds were solely calculated based on the peptide length (N) using $Rg = 2.54 * N^{0.522}$ for the upper and $Rg = (3/5)^{1/2} * 4.75 * N^{0.29}$ for the lower bound [1].

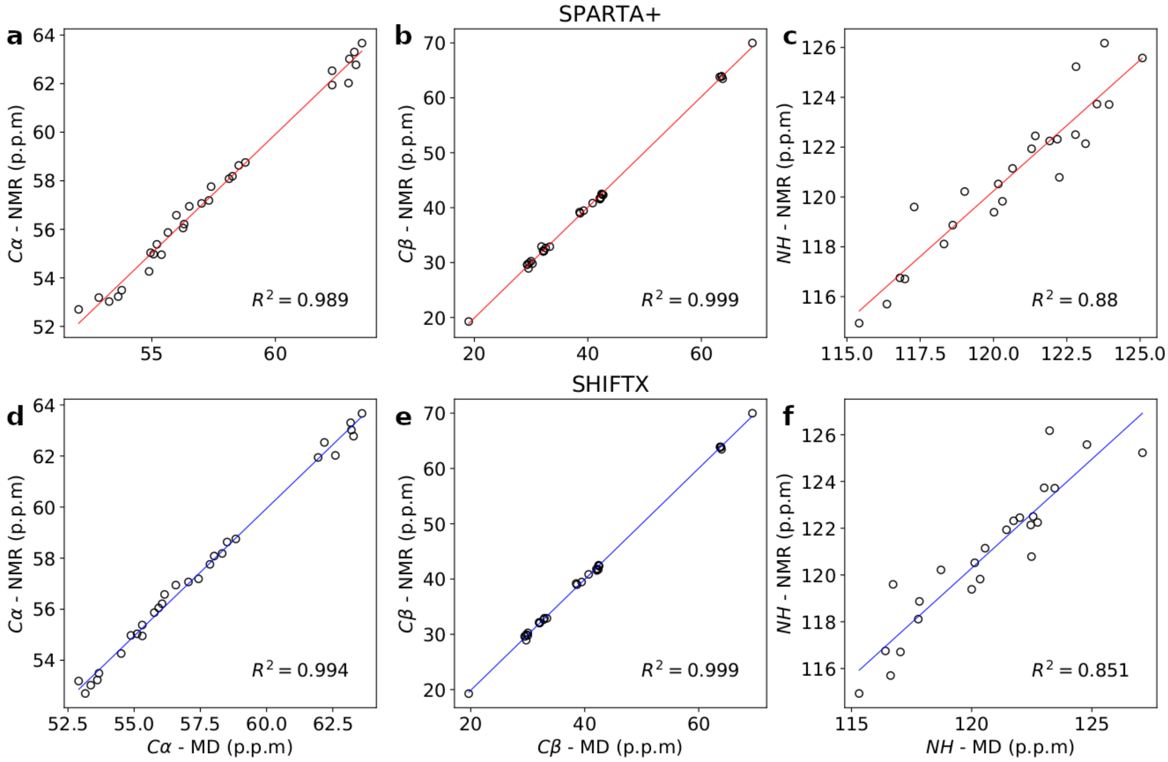

Figure 5: **Comparison against NMR data.** Chemical shift difference between MD-derived calculations for **a), d)** $C\alpha$, **b), e)** $C\beta$, and **c), f)** NH atoms and the experimentally measured for the N-terminal of p53 (Biological Magnetic Resonance Data Bank entry number 17760). Experimental data was obtained for the full length N-terminal domain (residues 1-96) but only data corresponding to residues 10 to 40 was used for the comparison. Calculations were performed with SPARTA+ (top row) and SHIFTX2 (bottom row).

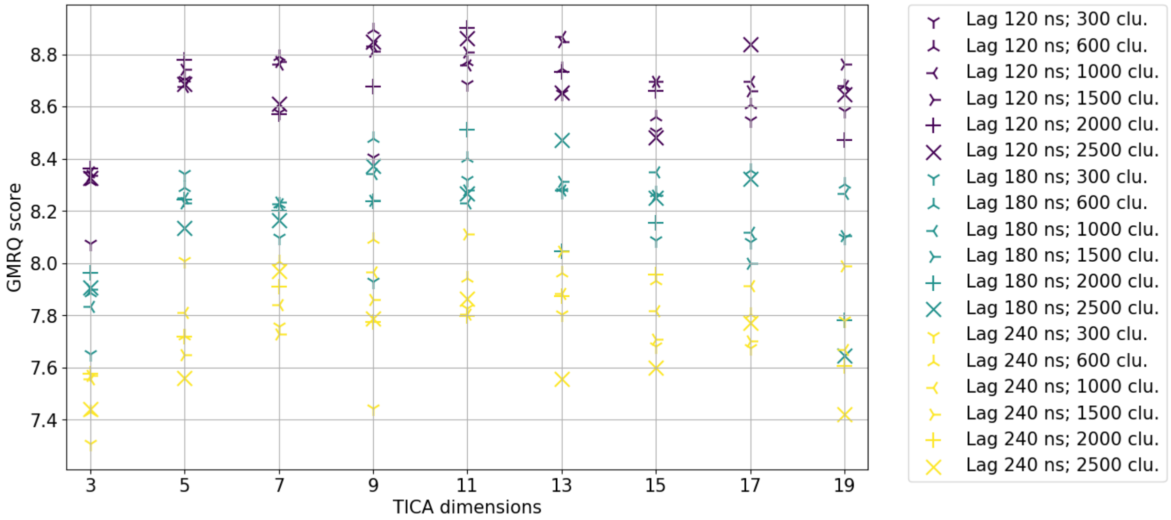

Figure 6: **Model selection.** The generalized matrix Rayleigh quotient (GMRQ) yields a score that allows to compare different MSMs built with the same data, hence it offers a way to perform parameter selection [2]. We computed $\sim$ 160 MSM with different number of TICA components (projected at a fixed lag time of 2 ns) and clusters. For each model we report the mean GMRQ value for 10 rounds of cross validation. The procedure shows similar trends across all lag times: the GMRQ peaks using 9, 11 or 13 TICA dimension. A final model with 9 TICA dimensions and 600 clusters was selected.

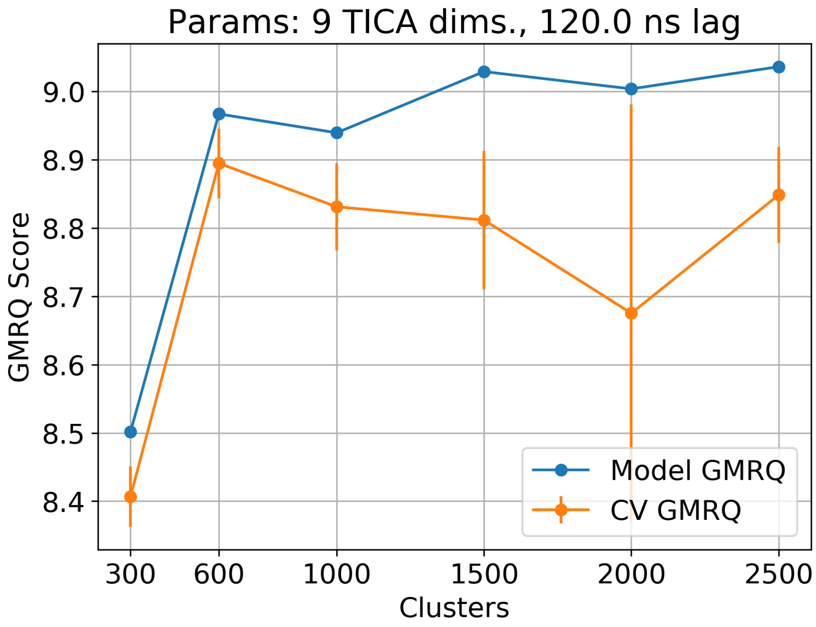

Figure 7: **Model parameters: cluster selection.** The effect of the number of clusters shows a maximum score with 600 clusters.

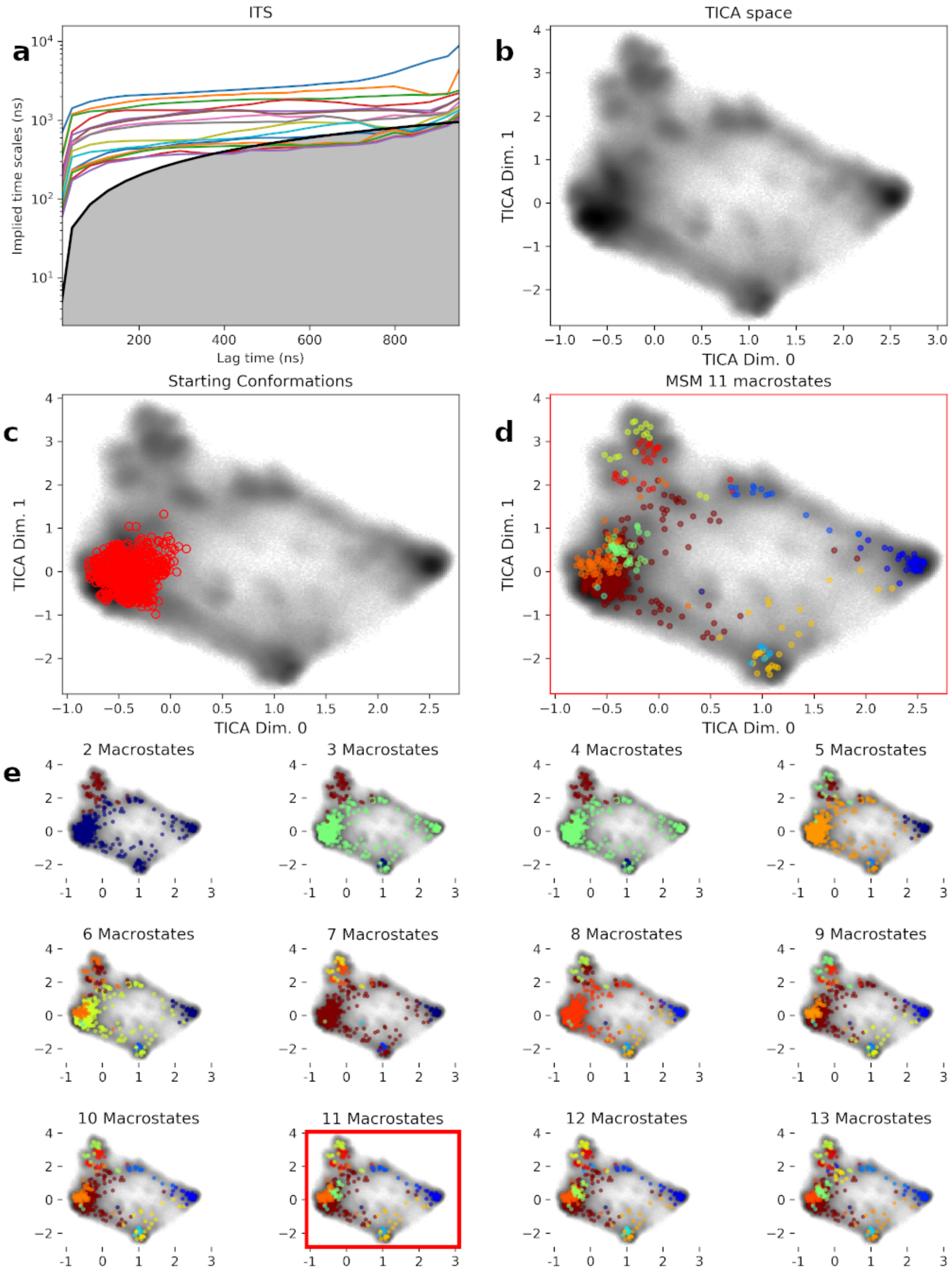

Figure 8: **Markov State Model** parametrization. **a) Implied times scales**. A final lag time of 120 ns was used to create the MSM. **b) TICA space** for the first and the second TICA dimensions. **c) Starting conformations** location on the TICA space. **d) Macrostate distribution** on the TICA space. Microstates are located based on their centers and color mapped following their macrostates assignment. **e) Discretization of the TICA space** by incrementing the number of macrostates. A final number of 11 macrostates was chosen.

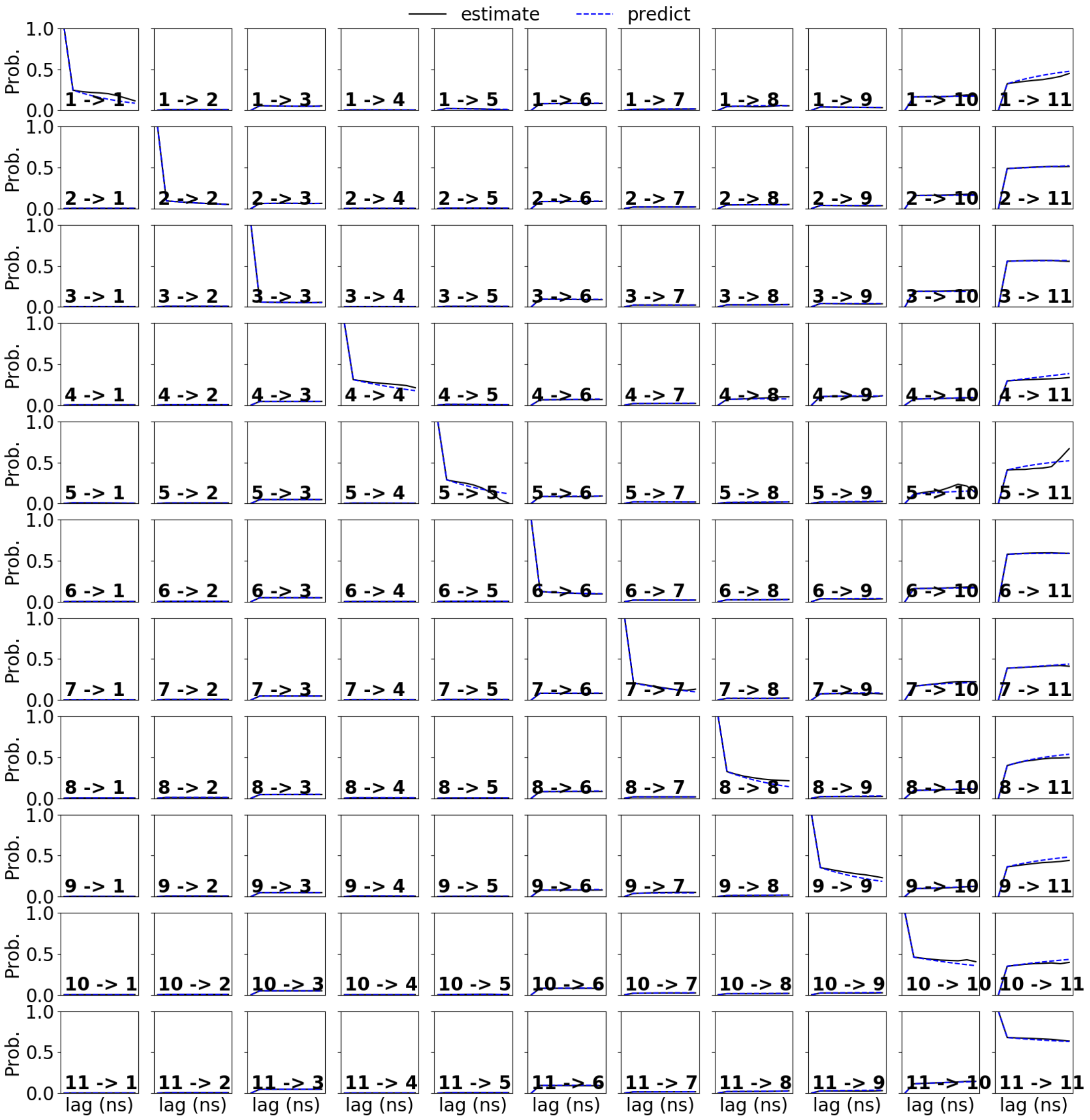

Figure 9: **Chapman-Kolmogrov test.** Chapman-Kolmogorov test shown for all transitions on the 600-microstate MSM. The Chapman-Kolmogorov test is performed to know whether the model make predictions (dashed lines) which are consistent with the data (solid lines).

References

[1] Deborah K Wilkins, Shaun B Grimshaw, Véronique Receveur, Christopher M Dobson, Jonathan A Jones, and Lorna J Smith. Hydrodynamic radii of native and denatured proteins measured by pulse field gradient nmr techniques. *Biochemistry*, 38(50):16424–16431, 1999.

[2] Robert T McGibbon and Vijay S Pande. Variational cross-validation of slow dynamical modes in molecular kinetics. *The Journal of chemical physics*, 142(12):03B621_1, 2015.
